# A Bayesian Account of Vocal Adaptation to Pitch-Shifted Auditory Feedback

**DOI:** 10.1371/journal.pone.0169795

**Published:** 2017-01-30

**Authors:** Richard H. R. Hahnloser, Gagan Narula

**Affiliations:** Institute of Neuroinformatics and Neuroscience Center Zurich, University of Zurich and ETH Zurich, Zurich, Switzerland; University of Sheffield, UNITED KINGDOM

## Abstract

Motor systems are highly adaptive. Both birds and humans compensate for synthetically induced shifts in the pitch (fundamental frequency) of auditory feedback stemming from their vocalizations. Pitch-shift compensation is partial in the sense that large shifts lead to smaller relative compensatory adjustments of vocal pitch than small shifts. Also, compensation is larger in subjects with high motor variability. To formulate a mechanistic description of these findings, we adapt a Bayesian model of error relevance. We assume that vocal-auditory feedback loops in the brain cope optimally with known sensory and motor variability. Based on measurements of motor variability, optimal compensatory responses in our model provide accurate fits to published experimental data. Optimal compensation correctly predicts sensory acuity, which has been estimated in psychophysical experiments as just-noticeable pitch differences. Our model extends the utility of Bayesian approaches to adaptive vocal behaviors.

## Introduction

Humans and animals can quickly adapt their behaviors to changes in the environment. For example, running barefoot or in shoes through a forest or on sand, all require different gait patterns that are effortlessly recruited when needed. Whether to adapt or not to an unexpected sensory event often depends on whether the latter is self-caused or not. For example, is the cracking sound of a piece of wood caused by one’s own foot step or that of a predator? In the latter case, running away may be a good option whereas in the former case doing nothing might be better. Optimal motor adaptation seems to suggest that the brain must determine the cause of sensory input [1] and the degree to which the input is commensurate with one’s own motor actions.

The problem of estimating self-caused sensory feedback becomes challenging when sensors are noisy, are subject to damage (e.g. hearing loss due to a ruptured eardrum), or undergo age-related degradation (e.g. presbyopia of the human eye). In order to deal with inaccurate sensory information during possibly imprecise movements, the brain must optimally integrate sensory information with motor plans by weighing the reliability of a given source of information, in a fashion similar to statistically optimal cue combination [2] or “causal inference” models in Bayesian cognitive psychology [1,3].

A nontrivial relationship between sensory error and motor compensation occurs in the visuo-motor domain: When humans perform arm reaching movements in the presence of force fields (proprioceptive error) or under visual rotations (visual error) that shift the trajectory of the hand away from the straight-line target, subjects partially compensate for the induced errors by selectively adapting the target reaches: large errors induce smaller relative compensation than small errors [3–8].

Another prominent example of partially compensated errors occurs during vocal control by auditory feedback. It has been shown that acoustic feedback has a strong influence on the adaptive control of human voice fundamental frequency: When fundamental frequency (or pitch) of vowels in speaking subjects is slowly shifted without their awareness (using earphones and bone oscillators), subjects compensate partly for the shift within tens of trials [9,10]. This compensation suggests that subjects interpreted the perceived shift to have occurred partially as the result of a production error that requires correction. As documented by a large body of literature, the percent compensation applied by subjects in such experiments declines monotonically with increasing shift in fundamental frequency [11–13].

Humans are not the only ones who compensate partially in such pitch-shift experiments. When pitch feedback into the ears of singing Bengalese finches is shifted using miniature headphones, birds compensate only partially for the shift, and the larger the shift the lesser the birds compensate [14]. All these experiments demonstrating a reduced compensation to large errors raise the possibility of a universal explanatory mechanism.

Two candidate arguments have been proposed to elucidate why both singing birds and humans compensate only partially for perturbed pitch. Liu & Larson argue that failure to correct for large-magnitude errors evidences a self-protection mechanism that prevents environmental sounds from exerting aberrant influence on vocal output [11]. Accordingly, humans subconsciously may interpret the perceived shift as resulting from a change in the environment that is not caused by them and thus needs no correction. Sober and Brainard [14] propose a somewhat different line of reasoning based on their finding that the amount of compensation is proportional to the overlap between the distributions of produced pitch and perceived pitch. They argue that sensory errors drive learning best when these fall within the range of production variability, explaining why large shifts outside the production range are ineffective in driving adaptation. Thus, the former explanation relates partial compensation to inferred external causes of the shift, and the latter relates it to constraints inherent to vocal learning. These explanations qualitatively differ in that the former argues for optimality of the adaptive behavior (there is no need to adapt to external causes), whereas the latter emphasizes a limitation of the adaptive system (constraint of learning). Our aim is to formalize the Liu and Larson’s proposal into a set of equations that can be used to fit data, similarly to Sober and Brainard’s model.

Computational models accounting for partial motor compensation have been previously proposed. Commonly, sensorimotor adaptation is explained using forward models [4,15]. Briefly, the idea is that the brain possesses an internal model of the system dynamics (e.g. hand position in a reaching task or fundamental frequency in a vocalization task) called the “forward model”. The forward model is able to generate an estimate of future sensory feedback based on a current motor command, hence the use of the term “forward”. If there are unexpected deviations between the estimate and the feedback, then motor commands are corrected dynamically in proportion to the perceived error [16,17]. Recently, [3] and [4] have extended this approach to account for the decreased compensation under large sensory errors by explicitly modeling the gain associated with integrating any particular sensory error as a decreasing function of the error (specifically, this appears through a sub-quadratic loss function).

We are taking a more principled approach to partial compensation in which we estimate the produced motor output (pitch in our case) based on a statistically optimal *linear combination* of expected and actual feedback which is then used (after non-linear weighting) to generate a “sensory error” for subsequent motor adaptation. The derived motor adaptation rule is quite similar to the Kalman filter, except that the gain term for the sensory error is obtained from a more principled perspective than in [4]. To test whether partial compensation is congruent with optimal behavioral strategies, we investigate a simple theoretical framework inspired by Bayesian theories of motor adaptation [8]. We show that published data in both humans and birds can be fit by a simple Bayesian decision model based on the idea that subjects respond optimally to the conflicting feedback, i.e., they adapt pitch up to an extent commensurate with known (or estimated) reliability of sensors and actuators. Our results suggest that both humans and birds behave optimally given knowledge of sensory, motor, and neural imperfections. And, in principle, given sufficient experimental data, the magnitude of these imperfections can be estimated from the data.

## Results

We present our work in the nomenclature of birdsong, which is our primary expertise. In a typical experiment, the **impinging pitch** *p*′ at the bird’s ears is the sum of the **produced pitch** *p* and the artificially applied **pitch shift** *p*_Δ_:

$$p_{\text{ear}}=p+p_{\Delta },$$

where pitch is reported in logarithmic coordinates of cents, *p* = 1200 log_2_ (*F*) − *κ*, with *F* the pitch (or fundamental frequency) in *Hz* and *κ* an arbitrary constant. Note that if birds do not wear pitch-shifting earphones then the impinging pitch *p*_ear_ at the ears equals the produced pitch *p*.

It is well known that the produced pitch *p* of a song syllable is not constant but it fluctuates from one rendition of the syllable to another. We approximate the distribution of produced pitch *p* across syllable renditions by a Gaussian with **mean (produced pitch)** *μ*_*m*_ and **variance (of produced pitch)** $\sigma_{m}^{2}$:

$$P(p)=N(\mu_{m},\sigma_{m}^{2}),$$

where *P*(*p*) is the probability of measuring the produced pitch *p* using a microphone in such an experiment and where *N*(*μ*, *σ*^2^) denotes a Gaussian distribution with mean *μ* and variance *σ*^2^. For the motor standard deviation of bengalese finch song syllables we take *σ*_*m*_ = 46 cents. We obtained this value by multiplying the median absolute deviation (MAD) of pitch distributions reported in [18] by 1.4826, which is the known factor that relates MAD and Gaussian standard deviation, i.e., *σ*_*m*_ = 31 * 1.4826 = 46 cents.

In the subsequent treatment, *μ*_*m*_ has the role of the bird’s current motor plan which together with the (constant) motor variability $\sigma_{m}^{2}$ is relayed to a sensory area as priors used for pitch estimation (we assume the sensory area has no information about the actual produced pitch *p*, only about its plan *μ*_*m*_, the sensory feedback *p*_*f*_, and the typical motor and sensory noise variances $\sigma_{m\;}^{2}$ and $\sigma_{f}^{2},$ respectively). In other words, we assume their perceptual system receives an internal expectation of pitch, which is not available when another bird sings. Evidence for such forward models (not of pitch per se, but of auditory input in more general terms) in both mammals and songbirds is provided by neurons in auditory brain areas that respond almost exclusively to feedback distortions [19,20]. We thus model pitch self-perception as the result of combined input from two separate sources, one being an internally expected feedback stemming from a forward model of the vocal organ and the other auditory feedback.

Given there must be noise in cochlear and neural processing of pitch, we assume the **auditory pitch feedback** *p*_*f*_ encoded in the brain’s auditory processing stream to be a Gaussian distributed random variable with mean *p*_*ear*_ and **variance (of pitch feedback)** $\sigma_{f}^{2}$:

$$P(p_{f}|p_{\text{ear}})=N\left(p_{\text{ear}},\;\sigma_{f}^{2}\right)$$

where *P*(*p*_*f*_|*p*_*ear*_) is the probability of observing pitch feedback *p*_*f*_ given impinging pitch at the ears *p*_*ear*_. Unfortunately, the current birdsong literature documents no attempts of measuring the variance of pitch feedback $\sigma_{f}^{2}$, which is why $\sigma_{f}^{2}$ is an unknown parameter in our model that we estimate through a simple fitting procedure described in Table 1. Given the two sources of noise, on average, birds try to reproduce a **pitch target** μ* they have acquired from a tutor during a sensory song learning phase [21]. When birds are not subjected to pitch-shifted feedback (*p*_Δ_ = 0), birds simply achieve a good copy of tutor song when the mean produced pitch equals the target, i.e., when *μ*_*m*_ = μ*. However, under pitch-shifted feedback (*p*_Δ_ ≠ 0), the mean produced pitch *μ*_*m*_ deviates from the target by an amount *ϵ*:

$$\mu_{m}=\mu *+\epsilon .$$

**Table 1:** Stochastic algorithm for finding the motor bias *ϵ* in response to a given pitch shift *p*_Δ_ and for identifying optimal model parameters $\sigma_{f}^{2}$ and *k* by fitting motor bias (compensation) data.

1.	Pick a pitch target μ* and choose model parameters $\sigma_{f}^{2}$ and *k* characterizing the bird’s sensory noise and perceptual priors
2.	Choose a feedback shift *p*_Δ_ (in cents), set the corrective bias to zero initially, ϵ = 0
3.	Draw a set of *n* (e.g., *n* = 200) random pitch samples *p* according to $N(\mu_{m},\sigma_{m}^{2})$ and associated random auditory feedback signals *p*_*f*_ according to $\;N(p+p_{\Delta },\;\sigma_{f}^{2})$ (assume the bird produces *n* renditions of the target syllable)
4.	Compute the one-source posteriors *P*(*s*|*p*_*f*_) and the pitch deviations Δ*p* (Eq 4) of these *n* renditions
5.	Change the corrective bias according to ϵ → ϵ − 0.001〈Δ*p*〉_*n*_ and return to Step 3 until convergence.
6.	Compute the percent compensation as *c* = 100|〈*ϵ*〉_*n*_|/*p*_Δ_, where the running average 〈*ϵ*〉_*n*_ runs over the last set of renditions
7.	Go to Step 2 and pick a new feedback shift *p*_Δ_.
8.	Evaluate the goodness of fit (compute the average fitting residual as a function of *p*_Δ_, as in Fig 2a and 2b). If the average residual is larger than in the previous fit, reject the latest parameter choices and proceed to Step 1 to pick new parameters $\sigma_{f}^{2}$ and *k* (randomly deviating from the current best choice by up to one order of magnitude).

That amount, the **corrective pitch bias** *ϵ* is our quantity of interest. For a given fixed pith shift *p*_Δ_ applied across many syllable renditions, we compute the equilibrium pitch bias 〈*ϵ*〉 based on optimality criteria, as detailed in the following paragraphs.

One goal of the perceptual system is to decide whether the feedback *p*_*f*_ is within the expected range (within the known motor variance $\sigma_{m}^{2}$) of the expected value *μ*_*m*_. The larger the sensory noise (the larger $\sigma_{f}^{2}$), the more likely will an imposed shift *p*_Δ_ be interpreted as being *self-caused*. By contrast, when the feedback *p*_*f*_ differs greatly from the expectation *μ*_*m*_, then such a difference cannot be reconciled and the perceptual system decides that feedback is contaminated with another bird’s vocalization or with some other environmental disturbance.

Thus, the critical problem is to estimate whether the feedback *p*_*f*_ is self-caused or not. The Bayes optimal approach is to compute the posterior probability of the source of a sensory event conditioned on the sensory input. Essentially, we can consider the source (S) of an event to be a Bernoulli random variable taking on one of two values *S* ϵ {*s*, *e*} denoting ‘self’ and ‘external’, with prior probabilities that satisfy *P*(*s*) + *P*(*e*) = 1. In this approach *P*(*s*|*p*_*f*_) is the posterior probability that the perceived pitch is ‘self’ generated and *P*(*e*|*p*_*f*_) that it is externally generated.

These posterior probabilities can be used to determine for example whether auditory input during a particular vocalization is self-caused, which is the case if *P*(*s*|*p*_*f*_) > *P*(*e*|*p*_*f*_). For example, a good adaptive strategy could be to retune the produced pitch when the auditory input is self-caused, *P*(*s*|*p*_*f*_) > 1/2, and its pitch is off target (not close to μ*). However, in the following treatment we will use Bayesian inference to retune pitch, based not on a decision about the source but instead on pitch estimates weighted by their posterior source probabilities.

According to Bayes’ theorem, we can write the posterior source probability *P*(*s*|*p*_*f*_) in terms of the likelihood *P*(*p*_*f*_|*s*) of observing the feedback *p*_*f*_ given it is produced by the bird, as follows:

$$\begin{array}{lll} P\left(s|p_{f}\right) & = & \frac{P\left(p_{f}|s\right)P\left(s\right)}{P\left(p_{f}\right)}=\frac{P\left(p_{f}|s\right)P\left(s\right)}{P\left(p_{f}|s\right)P\left(s\right)+P\left(p_{f}|\text{e}\right)P\left(\text{e}\right)}\\ & = & \frac{P\left(p_{f}|s\right)}{P\left(p_{f}|s\right)+\frac{P\left(p_{f}|\text{e}\right)P\left(\text{e}\right)}{P\left(s\right)}}\\ & = & \frac{P\left(p_{f}|s\right)}{P\left(p_{f}|s\right)+k} \end{array}$$ (1)

where *k* is a free parameter that depends on two factors: i) the unknown ratio of prior probabilities *P*(*s*) and *P*(*e*), and ii) the unknown pitch likelihood *P*(*p*_*f*_|e) given an external source. The parameter *k* is motivated by the idea that birds may have an accurate forward model of their own motor system but not of the world in general, which boils down to a uniform model and thus a single scalar parameter.

The likelihood *P*(*p*_*f*_|*s*) of (non-shifted, *p*_Δ_ = 0) pitch feedback can easily be computed assuming independence of sensory and motor noise sources:

$$\begin{array}{lll} P(p_{f}|s\text{)} & = & \int P\left(p_{f}|q,s\right)P\left(q\right)dq\\ P\left(p_{f}|s\right) & = & \frac{1}{\sqrt{2\pi \left(\sigma_{f}^{2}+\sigma_{m}^{2}\right)}}e^{-\frac{{\left(\mu_{f}-\mu_{m}\right)}^{2}}{2\left(\sigma_{f}^{2}+\sigma_{m}^{2}\right)}} \end{array}$$ (2)

Where we made use of the definitions $P(p_{f}|q,s)=\frac{1}{\sqrt{2\pi }\sigma_{f}}e^{-\frac{{\left(p_{f}-q\right)}^{2}}{2\sigma_{f}^{2}}}$ and $P(q)=\frac{1}{\sqrt{2\pi }\sigma_{m}}e^{-\frac{{\left(\mu_{m}-q\right)}^{2}}{2\sigma_{m}^{2}}}$ as previously introduced. The integral in Eq 2 arises from the fact that birds do not have access to the true produced pitch *p* but can only optimally infer its consequences by summing over all possibilities weighted by their probabilities. By inserting Eq 2 into Eq 1 we obtain a closed expression for the posterior probability *P*(*s*|*p*_*f*_).

To estimate the pitch discrepancy perceived by birds, we first consider the special case in which birds inferred the feedback stemming from one source, e.g. *P*(*s*|*p*_*f*_) = 1. In this case, the (optimally) **combined pitch** *p*_*opt*_ given the noisy measurement *p*_*f*_ and the prior *μ*_*m*_ is given by the 1-step iteration of a Kalman filter:

$$p_{opt}=argmax_{p}\left[P\left(p_{f}|p,s\right)P\left(p\right)\right]=\frac{\sigma_{f}^{2}\mu_{m}+\sigma_{m}^{2}p_{f}}{\sigma_{f}^{2}+\sigma_{m}^{2}}$$ (3)

This formula also corresponds to the optimal combination of two noisy sensory cues, see e.g. [2]. As expected, the combined pitch *p*_*o*_ always lies somewhere in between *p*_*f*_ and *μ*_*m*_. The inferred pitch discrepancy is Δ*p* = *p*_*opt*_ − μ*. Naively, the adaptation strategy could be to apply a pitch change *ϵ* such that the new combined pitch *p*_*opt*_ coincides with the target μ*. However, such strategy would be optimal only if birds were certain about the feedback being self-caused, which we assume is not the case. To take into consideration the posterior probabilities of self- versus external sources, we perform Bayesian inference [22] according to which the least-square pitch estimator is the conditional mean pitch, which provides us with the following expression for the perceived pitch deviation of a given syllable rendition:

$$\Delta p=P\left(s|p_{f}\right)p_{opt}+P\left(e|p_{f}\right)\mu_{m}-\mu^{*}=\epsilon +\left(p_{f}-\mu_{m}\right)P\left(s|p_{f}\right)\frac{\sigma_{m}^{2}}{\sigma_{f}^{2}+\sigma_{m}^{2}}$$ (4)

**Fig 1:**
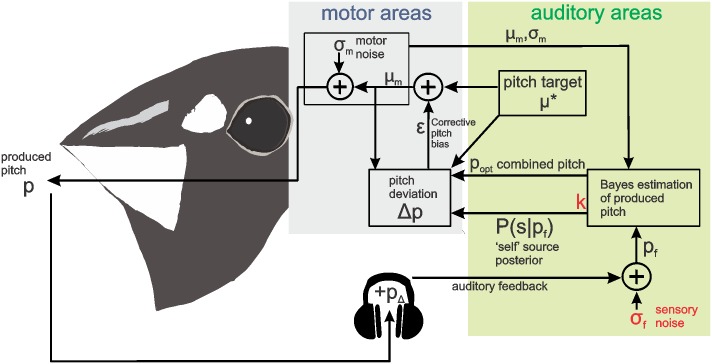
Model of optimal pitch adaptation. Motor areas in the brain generate a motor plan *μ*_*m*_ by integrating a desired pitch μ* and pitch adaptation *ϵ*. The produced pitch suffers from motor noise. Auditory areas optimally combine the motor plan with corrupted feedback *p*_*f*_, then reweight the estimate by the probability of feedback being self-caused *P*(*s*|*p*_*f*_) to produce a final pitch deviation Δ*p* relative to the desired pitch μ*. The two free parameters highlighted in red are estimated by fitting pitch compensation data from Bengalese finches and humans (Fig 2).

The remaining idea is that birds compute a motor bias *ϵ* such that on average (across syllable renditions), the pitch deviation vanishes, 〈Δ*p*〉 = 0. Unfortunately, because we cannot isolate *ϵ* in Eq 4 algebraically, it is not possible to provide a closed-form solution for the motor bias *ϵ*. To find this bias in practice, we iteratively change the corrective pitch bias *ϵ* (in small steps) with each set *n* of syllable renditions as follows:

$$\epsilon \to \epsilon -\eta {\langle \Delta p\rangle }_{n},$$

where *η* is a small constant, e.g. 0.001. Using this procedure, for a fixed pitch shift *p*_Δ_, the running average of pitch deviation 〈Δ*p*〉_*n*_ converges to zero and the running average 〈*ϵ*〉 converges to the corrective motor response we are interested in. A schematic of the entire model is presented in Fig 1 and the algorithm for finding the motor bias *ϵ* is described in Table 1.

**Fig 2:**
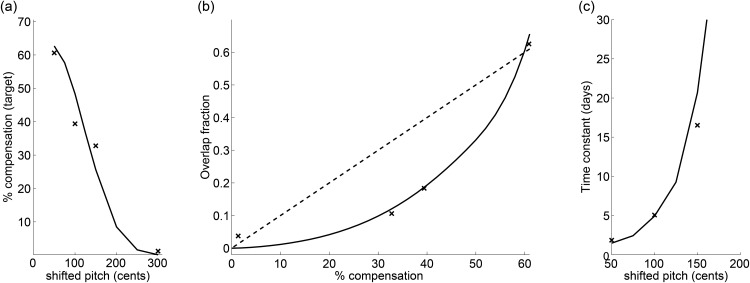
Model fits (black lines) to Bengalese finch data (crosses) digitized from [14]. Best fits to compensation data **(a)** and to overlap-fraction data **(b)** are achieved for *σ*_*f*_ = 23, *k* = 1.5 * 10^−4^. For comparison, the dashed line in (b) is the fit to the data provided by the overlap model in [14]. **(c)** The learning time constant (in days) was estimated as *τ* = *q*〈*P*(*e*|*p*_*f*_)〉/〈*P*(*s*|*p*_*f*_)〉, i.e. as the ratio of the self-versus external-source posterior probabilities (learning occurs mainly during inferred self-produced syllable renditions), *q* is a parameter estimated using a least-squared error fit.

The model comprising two parameters $\sigma_{f}^{2}$ and *k* summarized in Table 1 provides very good fits to Bengalese finch data that we manually digitized from [14], Fig 2.

**Fig 3:**
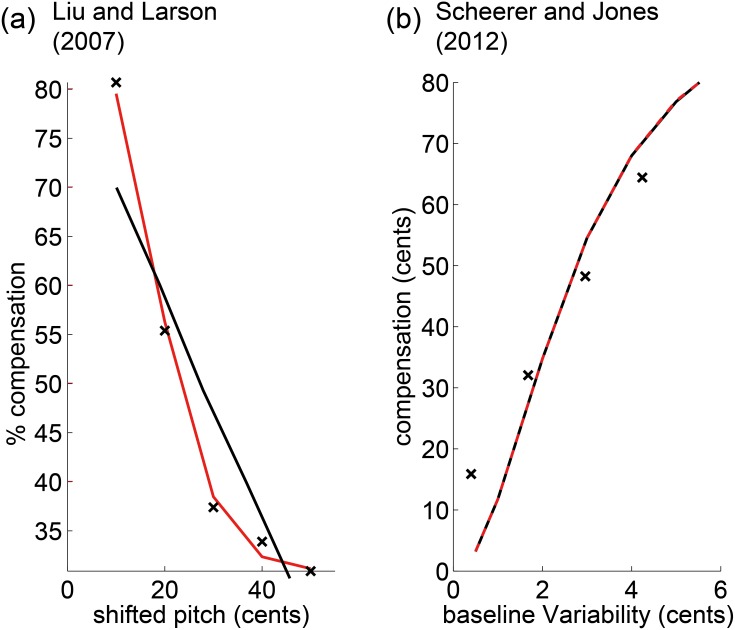
Model fits (lines) to human pitch compensation data (black crosses) digitized from [11]. **(a)** The model fit (black line) reveals only qualitative agreement but no precise match; *k* = 5.2 * 10^−4^, *σ*_*f*_ = 0 cents, *σ*_*m*_ = 32 cents. After introducing an additional offset parameter *ϵ*_0_ to account for a read-out bias, the model fit (red line) becomes excellent; *k* = 1.4 * 10^−3^, *σ*_*f*_ = 0 cents, *σ*_*m*_ = 14 cents, *ϵ*_*o*_ = 31 cents. **(b)** Fits (black line) through data points (crosses) extracted from the linear regression in [23]. *k* = 10^−320^ (essentially *k* = 0), *σ*_*f*_ = 7.5 cents. The same fit results (red dashed line) when enforcing a self-source interpretation, *P*(*s*|*p*_*f*_) 1. *σ*_*f*_ = 7.5. cents.

To test whether the model is also able to reproduce pitch adaptation in humans, we digitized the pitch-shift compensation curves in [11] and produced model fits using parameters *k*, *σ*_*f*_, and *σ*_*m*_ (motor variability was not reported in [11]). We found that model fits were good but not excellent, Fig 3a. We speculated that the main source of discrepancy between model and data could be the manner in which Liu and Larson quantified the motor response as the transient peak compensation (assessed relative to pitch traces in non-shifted control trials). Because of spontaneous pitch fluctuations, the peak transient compensation must be biased (any non-constant function exhibits local maxima that deviate from the mean), we argued, prompting us to introduce an additional bias parameter *ϵ*_0_ in model fits (*ϵ*_0_ is simply a constant bias added to *ϵ*). Indeed, including this additional parameter, model fits looked excellent, Fig 3a.

Humans and birds that produce more pitch baseline variability also compensate more to pitch-shifted feedback [18,23], an effect that we probed in our model. To investigate the precise dependence of compensation *ϵ* on pitch variability $\sigma_{m}^{2}$, we digitized the pitch-shift compensation curves as a function of pitch variability reported in [23] and fitted these curves using model parameters *k* and *σ*_*f*_. We found rough numerical agreement only in the range in which the parameter *k* was negligibly small, (Fig 3) implying that the model imposes an unconditional self-source interpretation, *P*(*s*|*p*_*f*_) ≃ 1. Perhaps not surprisingly, a self-source interpretation might have been enforced in [23] by telling participants to match a target note despite possible feedback alterations, which implies to reject the external-source interpretation, which in our model means to clamp the self-source posterior probability to one. Indeed, when we hard-coded *P*(*s*|*p*_*f*_) = 1 into the model, the equal fit resulted, Fig 3b (red dashed line).

**Fig 4:**
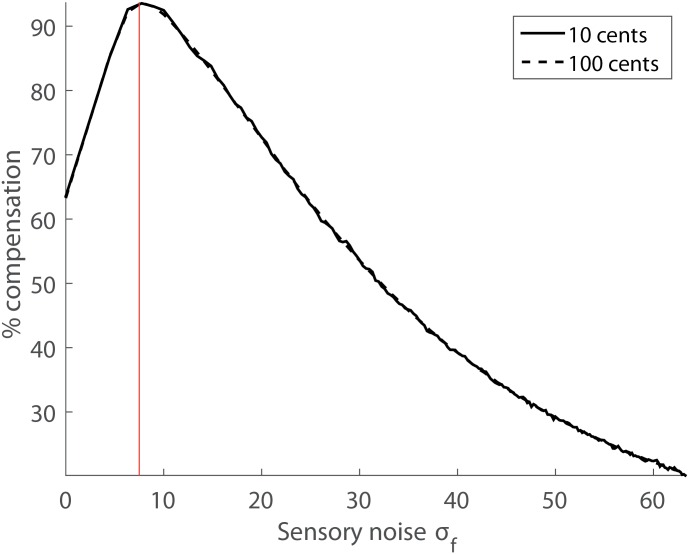
Non-monotonic dependence of percent compensation as a function of sensory noise. For both small and large pitch shifts *p*_Δ_ (superimposed full and dashed lines), the percent pitch compensation is a non-monotonic function that peaks at an intermediate level of sensory noise. Model simulations were performed with best-fit parameters for the human data in Fig 3: *σ*_*m*_ = 32 cents, *k* = 0. The red line marks the upper limit of our inferred pitch variability in humans (*σ*_*f*_ = 7.5 cents).

Interestingly, the model fits to [11] and [23], despite both applying to human data, differed in terms of their inferred sensory variability (*σ*_*f*_ = 0 cents vs *σ*_*f*_ = 7.5 cents). These numbers, however, were close enough and almost equally good fits resulted when we fitted [11] with *σ*_*f*_ = 7.5 cents inferred from [23]. Note that by contrast, the converse was not true and *σ*_*f*_ = 0 cents provided a poor account of the data [23]. Thus, overall we estimate *σ*_*f*_ in the range of 5–10 cents to provide a decent estimate of human perceptual pitch noise. A nontrivial prediction derived from our model is a non-monotonic dependence of the corrective motor bias as a function of sensory noise *σ*_*f*_, shown in Fig 4. Intuitively, the decreased motor compensation for large sensory noise *σ*_*f*_ arises from the disregard of noisy sensors by causal inference. By contrast, the decreased compensation for small noise *σ*_*f*_ is a result of trial-to-trial motor variability: Syllable renditions with pitch closer to the expected value *μ*_*m*_ have a larger posterior source probability than renditions with pitch further away from the expected value. Hence, when averaging over many syllable renditions, deviant renditions are more often ‘rejected’ from being self-caused than close ones; this rejection of outliers by causal inference is very prominent when sensory feedback is reliable.

## Discussion

We presented a normative model of vocal adaptation in birds and humans. The model contains only a small set of parameters, yet it reproduces the widely found decrease in motor compensation with increasing distortion of sensory feedback, and it also reproduces the recently demonstrated positive correlation between motor variability and motor compensation. We did not identify any serious disagreement with published data; any possible disagreement would be interesting, as it would introduce a requirement for model revisions.

Computationally, our model can be seen as a generalization of forgetting models of incomplete adaptation [24]: Namely, by replacing the term $P(s|p_{f})\frac{\sigma_{m}^{2}}{\sigma_{f}^{2}+\sigma_{m}^{2}}$ in Eq (4) by a constant term *α*, we find that the level of adaptation is set by two terms among which the target pitch μ* has the role of driving a forgetting term. Our nonlinear model is more general than simple forgetting-retention models in the sense that it correctly explains zero adaptation for highly magnified errors (Fig 2a) and that adaptation time constants are not fixed but depend on posterior evidence.

One of our aims was to include a model component for sensory processing, to support the possibility that motor adaptation might be constrained by the ability to perceive sensory feedback. No sensory component exists in overlap models of adaptive vocal behavior [18]. The model we studied includes sensory noise through the parameter *σ*_*f*_. The predicted compensation decreases with increasing sensory noise, and the model correctly produces zero adaptation (*ϵ* = 0 in Eq 4) in deaf birds (*σ*_*f*_ = ∞).

The pitch perception noise inferred in our model (*σ*_*f*_ = 1 to 7 cents) agrees well with reports in the literature. Human pitch perception studies report just-noticeable differences of 5–10 Hz for fundamental frequencies of complex harmonics [25] and of tonal speech [26]. If we assume a cumulative Gaussian function to underlie the measured psychometric functions, we obtain an estimated standard deviation *σ*_*f*_ of sensory noise of about 3–7 cents, agreeing well with our fit of the Scheerer and Jones (2012) data.

One way forward of using our modeling approach in further studies is to separately estimate perceptual pitch noise *σ*_*f*_ to further constrain the model down to essentially a single degree of freedom represented by the parameter *k*. In birds, for example, it would be interesting to probe the pitch discrimination ability using a go/no-go auditory discrimination paradigm [27] and to relate the perceptual precision of birds to their compensation magnitudes.

In principle, the dependence of motor compensation on sensory noise is non-trivial, Fig 4. Our model predicts that the compensation first increases as a function of sensory noise *σ*_*f*_ until it reaches a local maximum after which it decreases because the internal model becomes more reliable than the noisy auditory system. Unfortunately, this predicted non-monotonic relationship will not be simple to test in experiments, mainly for two reasons. First, it is not straightforward to model pitch noise. Second, even if it were possible to model pitch noise, our estimate of sensory variability *σ*_*f*_ = 7.5 cents in humans (Fig 3) coincides with the peak compensation in Fig 4. Because it is impossible to cancel out noise in the brains’ auditory processing streams, the low-noise region of the model seems to be inaccessible to experiments. Nevertheless, provided that our modeling approach applies to adaptation in other sensory systems such as vision, it may be possible to find behavioral paradigms in which highly precise sensors reside on the left of the non-monotonic compensation curve in Fig 4.

Regarding the speed of compensation there seems to be a discrepancy between humans and birds: in humans larger pitch shifts led generally to shorter adaptation latencies [11], which contrasts with birds in which large shifts are compensated more slowly than small shifts [14]. In humans, the speed of learning depends on many factors including environmental consistency [28]. Overall, given these discrepancies there is currently little hope to identify a unified principle underlying the speed of adaptation. For this reason we have excluded the time constant of compensation (Fig 2c) in the fitting procedure used to identify model parameters $\sigma_{f}^{2}$ and *k*.

It may be possible to further specify the role of model parameters from diverse reports in the literature. These are that singers compensate more than speakers [29] but good singers compensate less than untrained singers [30]. In the context of our model, singers compensating less than untrained singers might be explainable through *σ*_*m*_ being smaller in singers. By contrast, the same explanation via *σ*_*m*_ is unlikely to explain differences between singing and speaking. Rather, we would predict that the model parameter *k* is smaller during singing than during speaking, *k*_*sing*_ < *k*_*speak*_, possibly arising from increased attention to auditory feedback during singing (increase in *P*(*s*)). In summary, as suggested by our findings in Fig 3, we believe the model parameter *k* can account for instruction effects [31] according to which the compensation magnitude depends on the precise instructions given.

With regards to the parameter *k*, we have here assumed it to be a fixed constant combining self and external source priors *P*(*s*) and *P*(*e*), and the uniform distribution of other-produced pitch, *P*(*p*_*f*_|*e*). Had the fits presented in Fig 2 not looked convincing, we would have assumed that birds estimate the source priors as well as the pitch likelihoods and posteriors, for example via equalities *P*(*s*) = ∫ *P*(*s*|*p*_*f*_)*dp*_*f*_ and *P*(*e*) = 1 − *P*(*s*). When we played around with this possibility and iteratively estimated *P*(*s*) and *P*(*e*) in this way while estimating the fixed likelihood *P*(*p*_*f*_|*e*) as a free model parameter or even as a Gaussian function, we found that compensation curves (Fig 2a) looked much steeper than in reality, providing little preliminary support for such more extended models.

Motor adaptation is sensitive to the characteristics of the input modality. In humans, high fundamental frequencies of vocalizations are associated with lesser pitch compensation [11]. Whether these observations can be related deterministically to frequency-dependence of motor variability and sensory noise remains to be seen. Our approach could be used to model compensatory responses also with respect to other modalities such as sound amplitude. For example, [32] reported that for loudness-altered voice feedback, a compensatory gain close to 1 was only reported when subjects attempted to maintain a relatively quiet voice, but not when they produced louder vocalizations. In general, the Bayesian framework is powerful enough to be extended through the same normative principles to additional behavioral and sensory modalities.
